# Soil heavy metals contamination and health risk of an endemic plant in southeast of Damavand Mt., Iran

**DOI:** 10.1038/s41598-024-70819-3

**Published:** 2024-09-05

**Authors:** Maryam Naeimi, Parvaneh Ashouri, Samira Zandifar, Zohreh Boromand

**Affiliations:** 1 Desert Research Division, Research Institute of Forests and Rangelands, AREEO, Tehran, Iran; 2 Range Research Division, Research Institute of Forests and Rangelands, AREEO, Tehran, Iran; 3 Applied Geological Research Center of Iran, Nano-Bio Earth Lab., Karaj, Iran

**Keywords:** *Diplotaenia damavandica*, Endemic plant, Tehran, Phytoremediation, Heavy metals, Environmental chemistry, Ecology, Conservation biology

## Abstract

Considering the toxicological effects of some heavy metals (HMs) in which directly related to mortality and carcinogenicity in the population by their entrance from plants through livestock grazing, and medical skin cream, the rehabilitation of contaminated sites through phytoremediation by native plants might be quite challenging. *Diplotaenia damavandica* Mozaff. ex-Hedge & Lamond, is used as medical skin creams due to the existence of specific ingredients, which can be effective in treating skin disease. In the present study, the plant and associated soil sampling were performed around the boundary of *D. damavandica*. The concentration was measured using the Inductively coupled plasma mass spectrometry (ICP-MS). The results revealed the effect of existing endemic plants on reducing the average concentration of lead and zinc in soil by 40 and 60%, respectively, due to phytoremediation. EDX confirmed the presence of Pb and Zn in root and shoot tissues. Based on the results of this study, *D. damavandica* is an endemic perennial herbaceous plant with 60% biomass and prosperous root systems, which can grow in low contaminated areas of Pb in the southeast of Damavand Mt. Hence, the HMs pattern indicated less often in the aerial parts except for lead, which should be examined more carefully for skin cream uses.

## Introduction

**Fig. 1:**
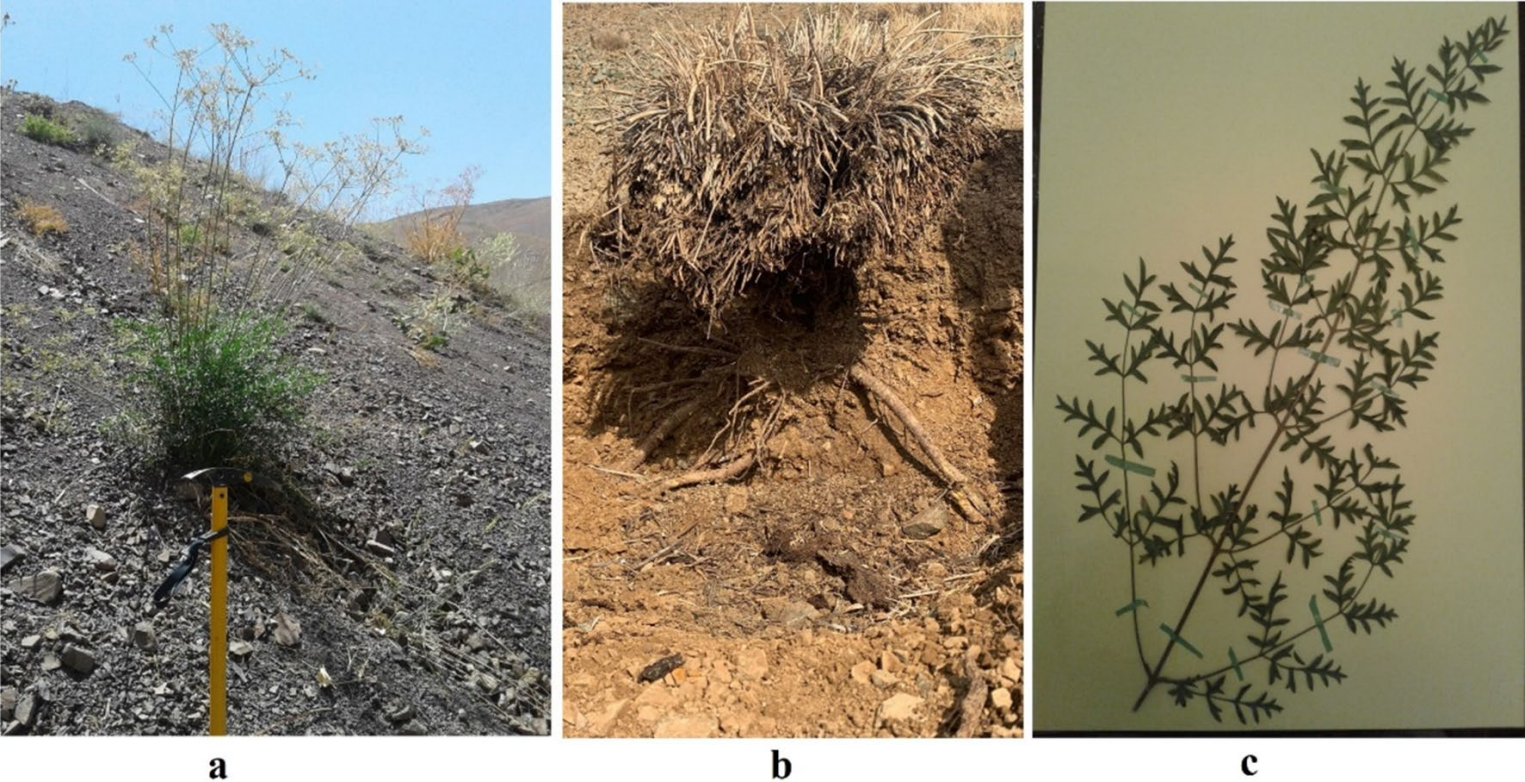
D. damavandica in the field (**a**) growing season, (**b**) dry season, and (**c**) a drawing of leaves in the herbarium of RIFR © 2024 by Maryam Naeimi is licensed under CC BY 4.0.

*Diplotaenia damavandica* Mozaff. ex-Hedge & Lamond (Gene bank association no. EU169259), from the Apiaceae family, known in local area as “Kozal”^1,2^, is an endemic specie (Fig. 1); Ref.^3^; in which both species of *Diplotaenia* have been investigated in their anatomy^4^ and aerial chemical composition^5,6^; while other aspects such as toxicology as well as environmental concerns have been poorly investigated. *D. damavandica* was practiced as medical skin creams due to the existence of ingredients such as xanthotoxin furanocoumarin and angelicin^7,8^, which can be effective in treating psoriasis, eczema, and vitiligo^9^. Moreover, during the growing season, it is a relative preference for grazing livestock^10,11^.

HMs unlike organic contaminants are generally immutable, not degradable, and persistent in soils^12^, as well as living organisms such as vegetation^13^. The concentration of HMs may be known as a biochemical action by passing from the roots to the shoots of plants^14,15^. The potential for HMs uptake and accumulation varies among different plant species, and the success of the phytoremediation process depends on the correct selection of the plant species^16,17^. Only a limited number of plants could be growing in HMs contaminated sites. In several studies, these plants have physiological adaptability, including metal tolerance strategies different from non-tolerant plants^18–20^. Phytoremediation by native rangeland plants^21,22^ has been indicated as an effective policy for soil remediation.

Considering the toxicological effects of some HMs on humans, especially Cd, Ni, Mn, and Pb, which represent a dangerous effect on biological systems, have been well documented^23,24^. Medical studies have shown that heavy metals are directly related to mortality and carcinogenicity in the population^25^. Heavy metals in soils are inaccessible to microorganisms due to their insidious and persistent nature, and can enter the human body through dermal contact and food chain intake, thereby endangering human health^25–27^. Particular toxic effects of lead were reported for the development of the brain and nervous system of kids, as well as long-term harm in adults, including increased risk of high blood pressure, cardiovascular problems and kidney damage^28–30^. Hence, due to the presence of volcanic soil in the study area as a primary source of HMs, there is a possibility of the entrance of HMs to human bodies in various paths such as skin creams^31^, livestock grazing and meat^32,33^, and dairy products^34^. Therefore, the presence of harmful HMs in the vegetation is a matter of concern which has not been investigated for *D. damavandica*.

In this study, it was aimed to investigate the values of HMs concentration in *D. damavandica* and associated soil, as well as the potential of the phytoremediation; Considering the point that it has been extensively practiced for medical skin cream. The limited existences literature impartial discussed the physiochemical characteristics and medical possibilities of *D. damavandica.* The present study stretches the highlights of warnings on the application of herbal medicines with the results of HMs in associated soil, aerial and root, through the possibility of phytoremediation.

## Materials and methods

### Study area and data collection

The study area was the habitat of *D. damavandica,* located in the eastern parts of Damavand City, Tehran Province, Iran (33° 54′ 56" N, 46° 04′ 30" E). *D. damavandica,* with the locality in Elburs Mts.^35^, north of the Damavand city, Iran (*plants.jstor.org*, 2010), with the altitude of above 2450 m.

**Fig. 2:**
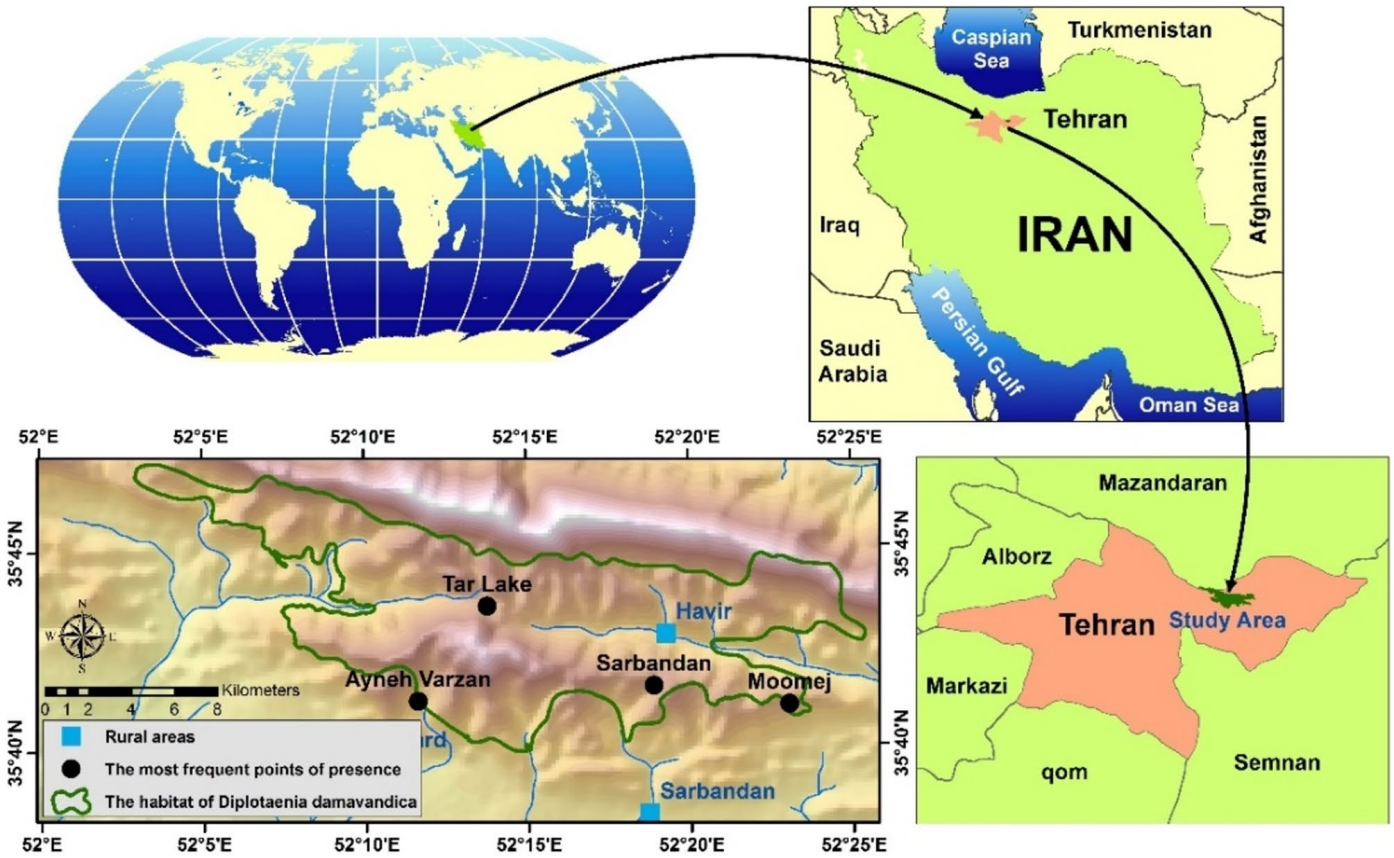
Location of the study area in the country, along with the ecological boundary of D. damavandica © 2024 is licensed under Creative Commons Attribution 4.0 International (created by ArcMap 10.5).

The results of the field survey confirm the existing sources indicate that the area of Kozal habitat covers about 17,654 ha in diverse geographical directions. The geographical location of the study is from 52° 03' to 52° 26' N, from 35° 40' to 35° 50' E, and the altitude is above 2400 to 3600 m. The mean of precipitation and temperature is 419 mm and 5 °C, respectively. Additionally, as shown in Fig. 2, the four locations with the highest frequencies of *D. damavandica* were discovered namely Tar-Lake, Momej, Sarbandan, and Ayenehvarzan, on the south aspect, slope of 20 to 60%, and altitude above 2450 m^36^.

Sampling was performed in late June of 2022. The soil and plant sampling was performed based on a systematic randomized design in four different geographical directions around the ecological habitat of *D. damavandica*. Note that the permission to collect *Diplotaenia damavandica* Mozaff. ex-Hedge & Lamond were obtained through the approval of the project in RIFR with the number of “2-09-09-020-980433”; Voucher specimens were deposited in a RIFR herbarium and was collected from the area as addressed above. Moreover, the information on the voucher specimen was identified by Dr Mozafarian which the name of the species is after him.

**Fig. 3:**
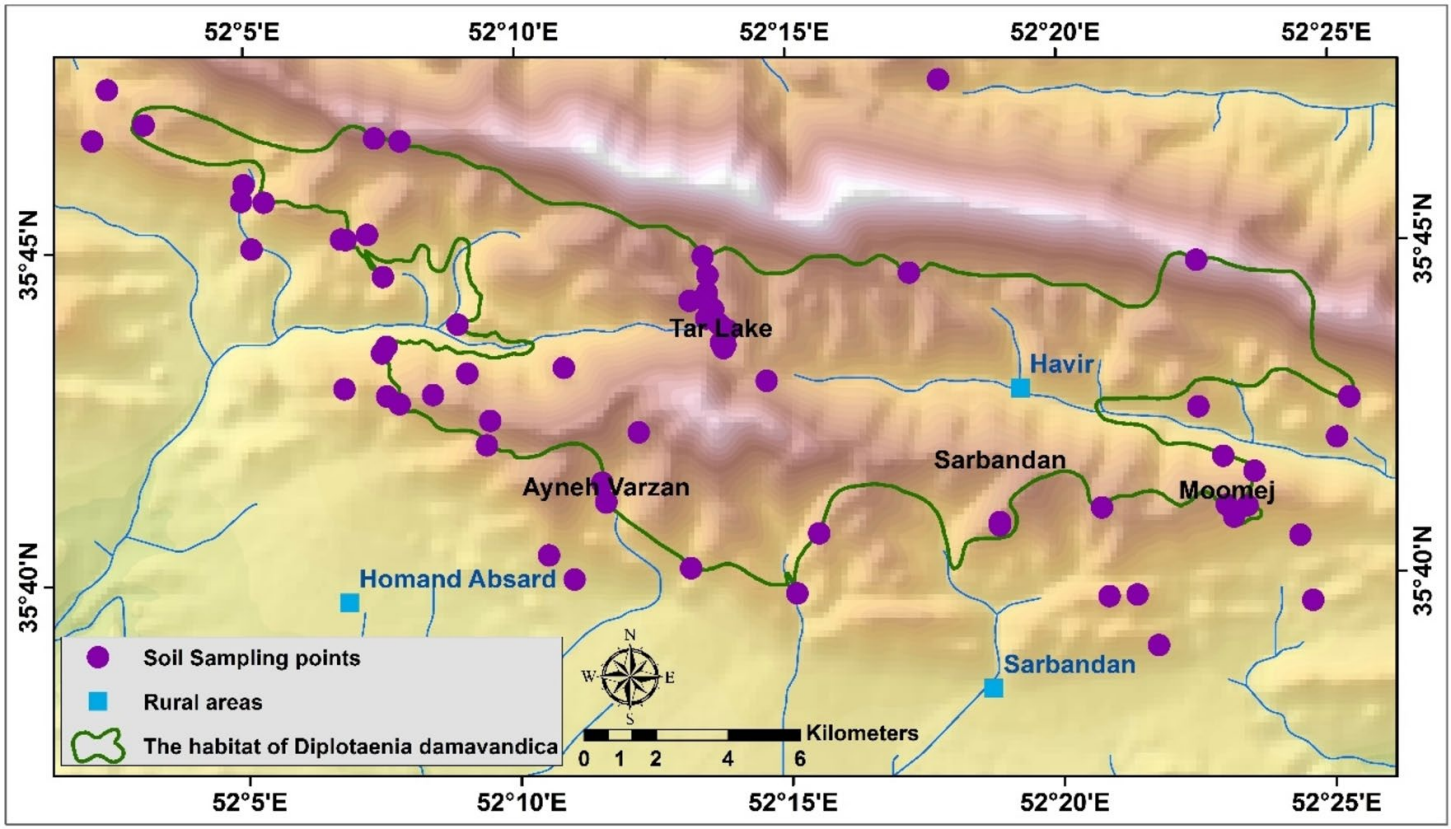
Soil sampling in the habitat of D. damavandica and control areas © 2024 is licensed under Creative Commons Attribution 4.0 International (created by ArcMap 10.5).

Soil samples were taken from 0 to 20 cm depth in rhizosphere. A total of 50 samples of soil and 10 samples of plants were collected (Fig. 3) and were sent for HMs analysis. It is necessary to mention that the sampling of the plant and soil was chosen considering same ecologically and edaphically conditions such as same plant species and soil characteristics.

In order to perform statistical comparisons, soil samples were collected outside the ecological habitat in similar edaphic and topographic conditions (as the control samples).

### Soil and vegetation analysis

After sampling, the root, shoot, and soil samples were placed in special envelopes and transferred to the laboratory. Plant samples were washed with DI water, and then oven-dried at 105°C for a day., while soil samples were air dried for 20 days. Later, plant and soil samples were ground to pass through no. 200 mesh sieve.

The total concentrations of Cd, Co, Cu, Mn, Ni, Pb, and Zn in the plant and soil materials, in the form of total concentration of cations, determined by the Inductively coupled plasma mass spectrometry (ICP-MS) analytical technique.

Soil samples were prepared through four acid analysis method. A near total digestion using multi acid digestion including HF, HCl, HClO_4_, and HNO_3_ with the scheme code MMS-01 and the Standard Practice ASTM D4698-21 for total digestion of sediment samples.

Regarding the plant samples, the ENV-02 ICP-MS analysis and microwave acid digestion of the samples with concentrated acid and oxidizer were carried out. Zero point two five grams of the ground sample was weighed into the digestion vessels, then 5.0 mL of HNO3 and 2.0 mL of H_2_O_2_ were added to each sample. Samples were digested until a clear solution was achieved. The entire digest was transferred quantitatively into 100 mL volumetric flasks using Milli-Q UPW, then stored in plastic bottles with caps on until the analysis process.

Continuously, soil pH and EC was determined in a soil/water solution with a volume ratio of 1:1 using pH and EC meter. Additionally, organic matter (%) and soil texture measured by Walkley–Black and hydrometer methods, respectively.

### Phytoremediation efficiency indices

For all collected dominant plants and each total concentration of metal, the bioconcentration factor (BF), the biological absorption coefficient (BAC), and the translocation factor (TF) was determined^37^. The dry or bulk density of typical soil in habitat (clay loam) was calculated as 1.28 g/cm3.

1 $$\text{BF }=metal \,concentration\, in\, shoots/metal\, concentration\, in \,soil$$

2 $$\text{BAC }=metal \,concentration\, in\, roots /metal \,concentration\, in \,soil$$

3 $$\text{TF }=metal\, concentration\, in \,shoots/metal\, concentration\, in \,roots$$

Later, the collected data were analyzed using SPSS 22 software, the Kolmogorov–Smirnov test for normality, and the Levene test for homogeneity of variance with (p maximum allowable concentrations). Then, the independent samples t-test analysis method was selected to perform statistical analysis and evaluate important differences. Data were analyzed using statistical significance at a significance level of p < 0.05.

### Scanning electron microscopy

FESEM and EDX were done for the confirmation of HMs presence in the associated soil as well as root and aerial parts of *D. damavandica*. The samples were prepared through the equestrian in 0.5 M phosphate buffer (pH 7.1–7.4) containing 2.5% of glutaraldehyde and kept overnight at room temperature and dehydrated with ethanol^38^.

## Results and discussion

### Soil properties

**Fig. 4:**
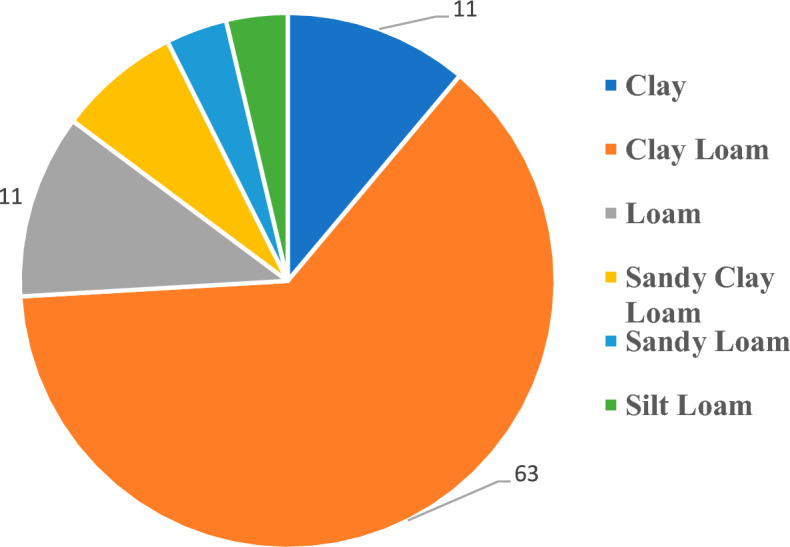
The percent and type of soil texture in the soil samples collected from the habitat of *D. damavandica*.

Figure 4 shows that *D. damavandica* likely grows in various types of soil, while the majority of soil types of its habitat are from heavy-clay soil, specifically clay loam which resulted in the ions accumulation characteristics.

Continuously, the comparison of the average of soil particles in the habitat of *D. damavandica* and control areas using the student's t-test demonstrated that the silt parameter has significant differences (Table 1). Regarding the pH, some researchers have found pH values of 7.6 to 7.85 on abandoned roofs in the Ubazi Gold Mine as well as Ilam bituminous mine, similar to this study findings^13,39^.

**Table 1:** Comparison of student's t-test of soil parameters in the habitat and control.

Parameter	Average (Control)	Average (Habitat)	T value	Significance
Sand (%)	4.14	2.03	0.094	ns
Clay (%)	2.47	1.76	0.725	ns
Silt (%)	3.34	1.59	0.045	*
EC (ds/m)	0.05	0.03	0.458	ns
pH	7.62	7.61	0.475	ns

*: The differences between habitat and control areas are significant.

**Fig. 5:**
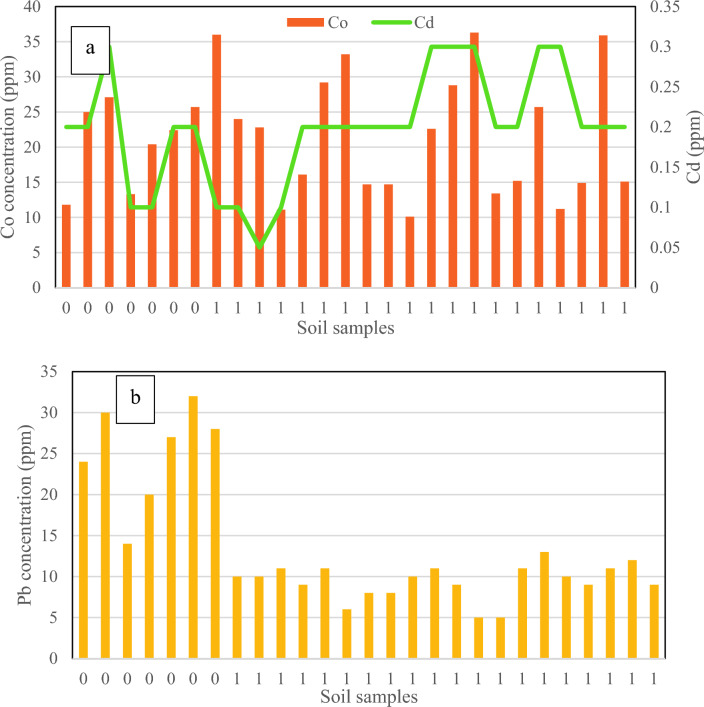
The results of (**a**) cadmium and cobalt, and (**b**) lead, in the habitat of *D.damavandica* and control areas in the horizontal axes as “0 & 1” respectively.

**Fig. 6:**
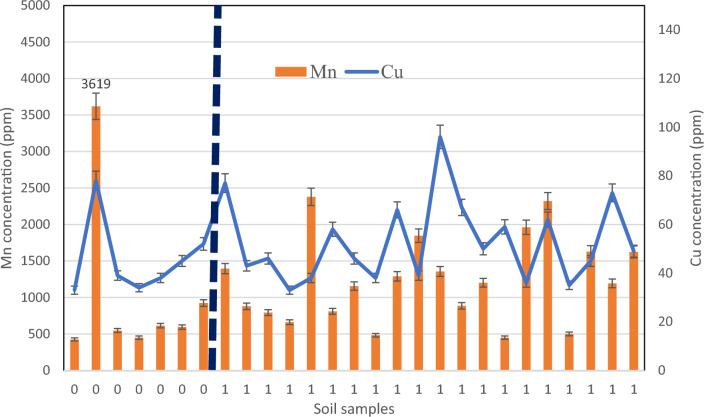
The results of manganese and copper in the habitat of *D.damavandica* and control areas.

**Fig. 7:**
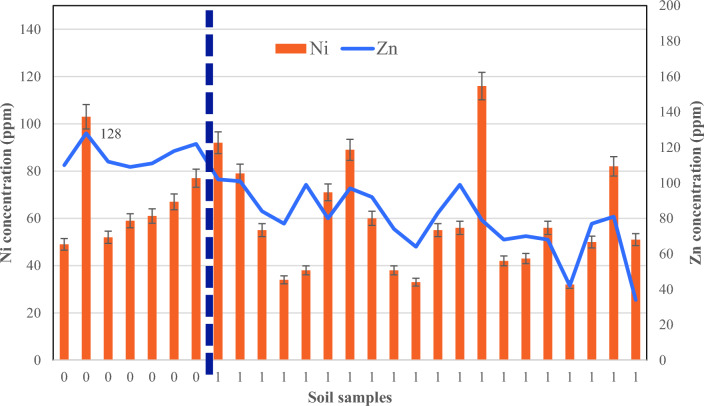
The results of nickel and zinc in the habitat of *D.damavandica* and control areas.

In addition, considering the presence of Mount Damavand, volcanic soil^40,41^, and the presence of heavy metals in such condition^42^, the total concentration of HMs was investigated in the associated soil of *D.damavandica,* then compared to the control areas in and out of the habitat. The survey of surface soil contamination (0–20 cm) around the roots of *D.damavandica* and the control areas (without the presence of the plant) presented in Figs. 5, 6 and 7. It must be noted that the border (black dot) of control samples (0) and habitat (1) is shown in the following figures; direction from the arrow to the right side of the horizontal axe, the frequency of the plant increases; while from the arrow to the left side the control areas get further to the habitat.

The results of cobalt and cadmium in Fig. 5 revealed no special trend between habitat and control areas. The maximum and minimum total concentration of cobalt in the control area was equivalent to 27 and 13 (ppm), respectively; while in the habitat, the highest and lowest cobalt concentrations were 36 and 10 (ppm), respectively, which indicates the plant's compatibility. The maximum and minimum total concentration of cadmium in both areas was found equivalent to below 0.5 (ppm), respectively.

Regarding the lead, as Fig. 6 and Table 2 demonstrated, a significant decreasing trend in the presence of *D. damavandica* can be stated. The highest and lowest lead total concentrations in the associated soil of *D. damavandica* were 28 and 1, respectively. Hence, 60% reduction on lead total concentration was observed.

**Table 2:** HMs data analysis and average data comparison using Student's t-test.

HMs	Soil standard^1^	Soil contamination standard^12^	Mean^1^ (Control)	Mean^1^ (Habitat)	T value	Significance
Cd	0.01–2	5	0.19	0.20	0.602	ns
Co	1–40	50	20.81	21.55	0.845	ns
Pb	10–15	100	25.00	9.40	0.000	*
Cu	2–25	500	45.57	52.80	0.327	ns
Mn	100–400	–	1024.29	1240.15	0.524	ns
Ni	10–50	530	66.86	58.60	0.399	ns
Zn	10–100	500	115.71	78.55	0.000	*

^1^mg/kg and ^2^pH > 7 for forests and rangelands, Iran’s Department of Environment, 2011.

*: The differences between habitat and control areas are significant.

Results of the total concentration of manganese and copper indicated no specific trend in the control areas and habitat (Fig. 6). Nevertheless, results show the capability of the endemic plant to coexist with manganese and copper concentration.

The nickel results indicated no particular trend between habitat and control areas (Fig. 7). The average nickel total concentration in the control and habitat area was equivalent to 66.86 and 58.60 ppm, respectively.

The analysis of zinc, according to Fig. 7, showed a distinctive trend between the control areas and the habitat. Yet, the higher values of zinc in areas further away from the habitat. It is necessary to note that the further the distance from the habitat, the zinc total concentration reaches 128 ppm; While, in the habitat, the average concentration is 78.55 ppm. Therefore, the endemic plant reduced the zinc average concentration by 40% due to phytoremediation.

HMs concentration in either habitat or control areas is below the standards of soil contamination (Table 2). However, the excessive concentration of Cu, Mn, Ni, Pb, and Zn in control areas compared to habitat was revealed compared to soil standard; while the significant mean comparison resulted for Zn and Pb. In addition, the Zn concentration of control areas (115 ppm) is higher than the soil standard (100 ppm). Interestingly due to the presence of *D. damavandica,* the concentration of both elements showed significantly lower.

### Phytoremediation efficiency indices

**Fig. 8:**
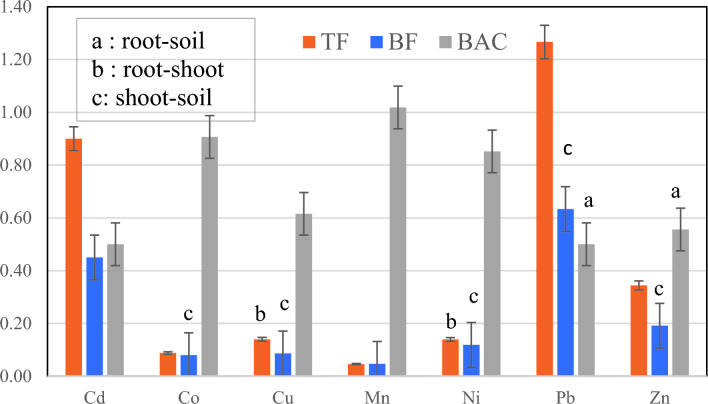
Comparison of calculated indicators including TF, BF and BAC of *D. damavandica* (a, b and c represent the significance of factors as presented in the chart).

The capability of the *D. damavandica* in phytoremediation of soil HMs calculated using the average of indicators including BF, BAC, and TF as shown in Fig. 8.

Overall, BF values were < 1 for all toxic metals (Fig. 8). Results of the calculated phytoremediation indicators confirmed that *D. damavandica* had relatively higher TF values than BFs for the same metal; TF values for Pb were > 1 indicated the phytoextraction of the plant. Previous studies also showed the successful removing of PB through extraction in *Tamarix smyrnensis*^43^, *Armeria arenaria*^44^, *Sesbania exaltata*^45^, and *Pelargonium hortorum*^46^ from contaminated soils. Note that, Continuous phytoextraction uses endemic plants with natural abilities to accumulate high contents of HMs (hyper-accumulators). Moreover, TF < 1 and BAC > 1 indicated the capability of Phytostabilization^47^. Hence, in the present study, *D. damavandica* is capable of phytostabilization of Mn. In the suitable plants for phytoremediation, the concentration of elements in the aerial parts is higher than those of the soil^48,49^.

The results illustrated the significance of the mean of Zn and Pb in both root-soil, plus shoot-soil (Fig. 8); while root-shoot showed the significance of Cu, Mn, and Ni. Additionally, shoot-soil mean comparison showed the significance of Co, Cu, and Ni.

The results indicated that except for Mn and Pb, other HMs in aerial tissues were downward compared to the associated soils and followed the pattern of soil > root > shoot.

Interestingly, the pattern for Mn is followed by root > soil > shoot, while the pattern for Pb is soil > shoot > root. Hence, the excessive amount of Pb in the shoot of *D. damavandica* makes the TF higher than other elements. Previous studies also reported the higher concentrations of Cd, Mo, and Pb that were found in the aerial parts of *Hordeum bulbosum L*., indicating the potential ability of phytoremediation^13^. Likewise, Pb accumulation in *Atriplex sp.* shoot, and root dry weight was also previously reported^50^.

Moreover, it was shown that *Achillea willhelmsii, Stipa barbata, and Aconthophyllum microcephallam* had the highest concentration of HMs uptake with concentrations of 103.7, 237.3, and 0.9 mg/kg, for Pb, Zn, and Cd respectively, and are good options for refining soil^51^.

**Fig. 9:**
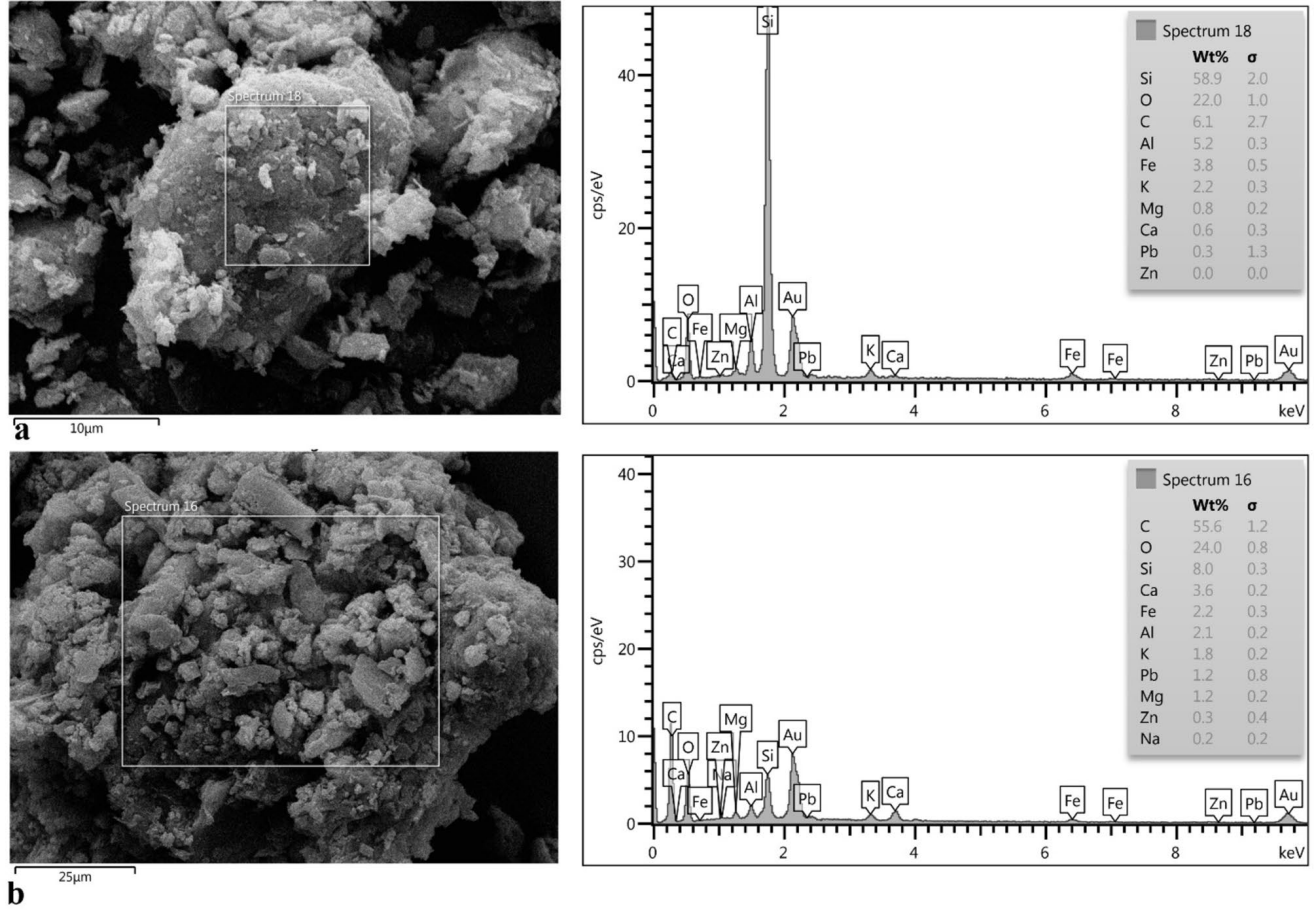
The FESEM-EDX of (**a**) associated soil, and (**b**) roots and aerial parts of *D. damavandica.*

Continuously, associated soil sample as well as root plus aerial parts of *D. damavandica* were observed by field emission-scanning electron microscope (FE-SEM) equipped with an energy dispersive spectrometer (EDX) detector as showed in Fig. 9. The results confirmed the presence of Zn and Pb ions in the roots and aerial parts of *D. damavandica.* Pb and Zn presence in the EDX spectrum in Fig. 9b confirmed the presence of Pb in the root and shoot tissues.

Moreover, the accumulation of Pb on the leaves of the street vegetation has been found^52^; while Pb and Zn reported in the roots and aerial parts of *D. damavandica* as showed in Fig. 9 through FESEM analysis. Other species such as *Commelina communis*, *Chrysanthemum indicum* and *Phytolacca acinosa* also tended to accumulate higher concentrations of Mn in the aerial than roots, presenting relatively high metal transport ability^18^.

Providing the argumentation that the highest phytoremediation efficiency in low to moderate areas due to the toxicity of HMs in plants with extensive root system and higher biomass^53^, the results of the present study justified for *D. damavandica*. On the other hand, *D. damavandica* was practiced as medical skin creams and due to the results of the present study the safety of herbal medicine must has been highlighted.

## Conclusions

Identification of plants in each area provides a better understanding of restorable natural resources and their applications. The results of the present study strongly confirmed the phytoremediation potential of *D. damavandica*, a grass specie belonging to the Apiaceae family. The HMs pattern indicated less often in the aerial parts except for lead. The confirmation of Pb in plant tissues was confirmed through FESEM-EDX. Hence, the excessive amount of Pb in shoots makes the TF higher than other elements, which gives the ability of phytoextraction. Given that the products of *D. damavandica* enter the food chain of the people of the region due to the entry to the livestock’s, dairy products and other human being by entering in the skin care creams, it is necessary to conduct HMs analysis, specifically more carefully for skin cream uses.

## Supplementary Information

Supplementary Figures.

## Data Availability

All data generated or analyzed during this study are included in this published article and the Supplementary File.
