# Supplementary material for: Soil heavy metals contamination and health risk of an endemic plant in southeast of Damavand Mt., Iran
Source: Sci Rep. 2024 Sep 5;14:20661. doi: 10.1038/s41598-024-70819-3 (PMC11377443; doi:10.1038/s41598-024-70819-3)
Supplement: Supplementary file 1 — Supplementary Figures. [file 41598_2024_70819_MOESM1_ESM.docx]

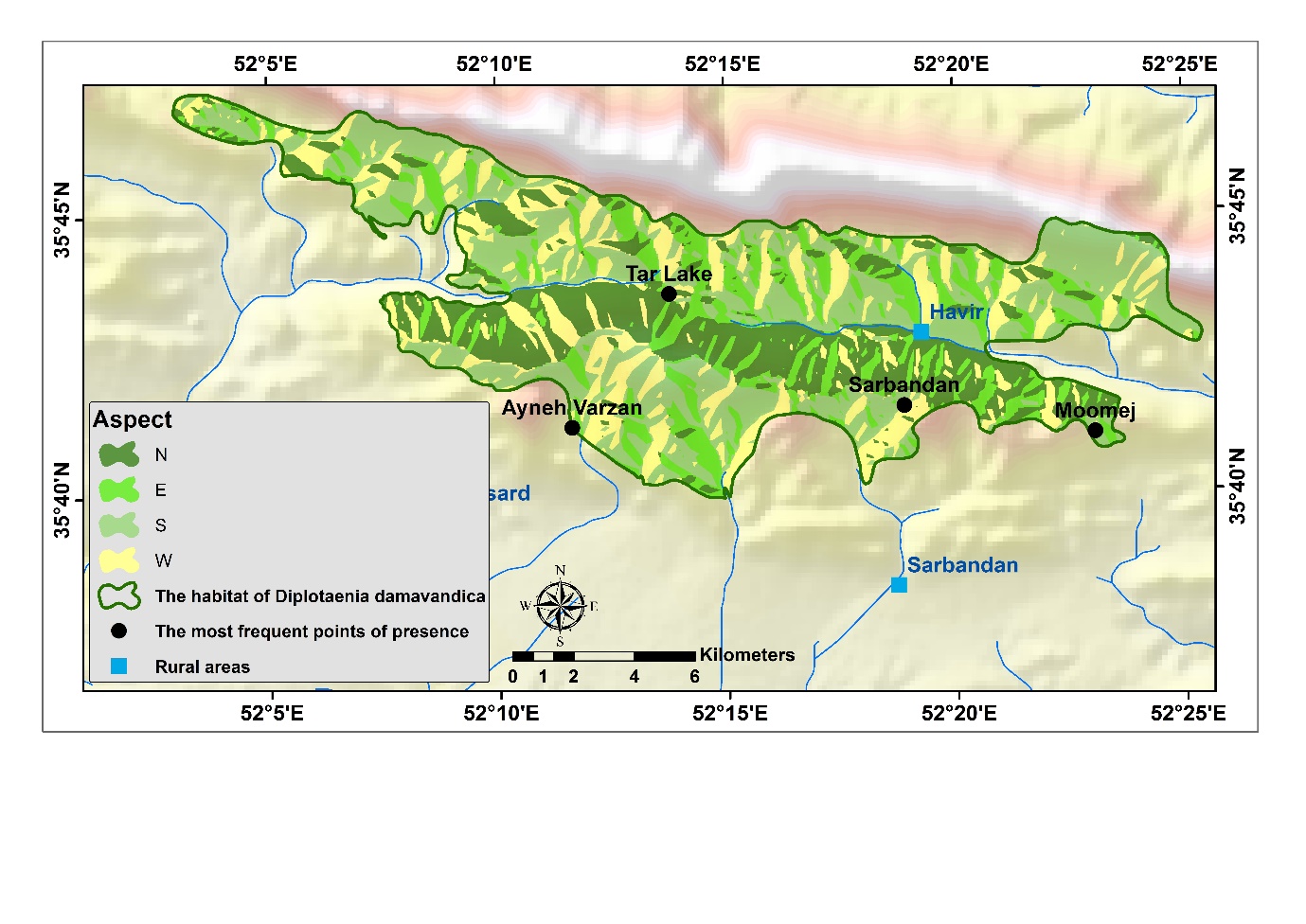


Fig. a. Fig. a. The aspect map of the study area © 2024 by Parvaneh Ashouri is licensed under CC BY 4.0 (created by ArcMap 10.5)


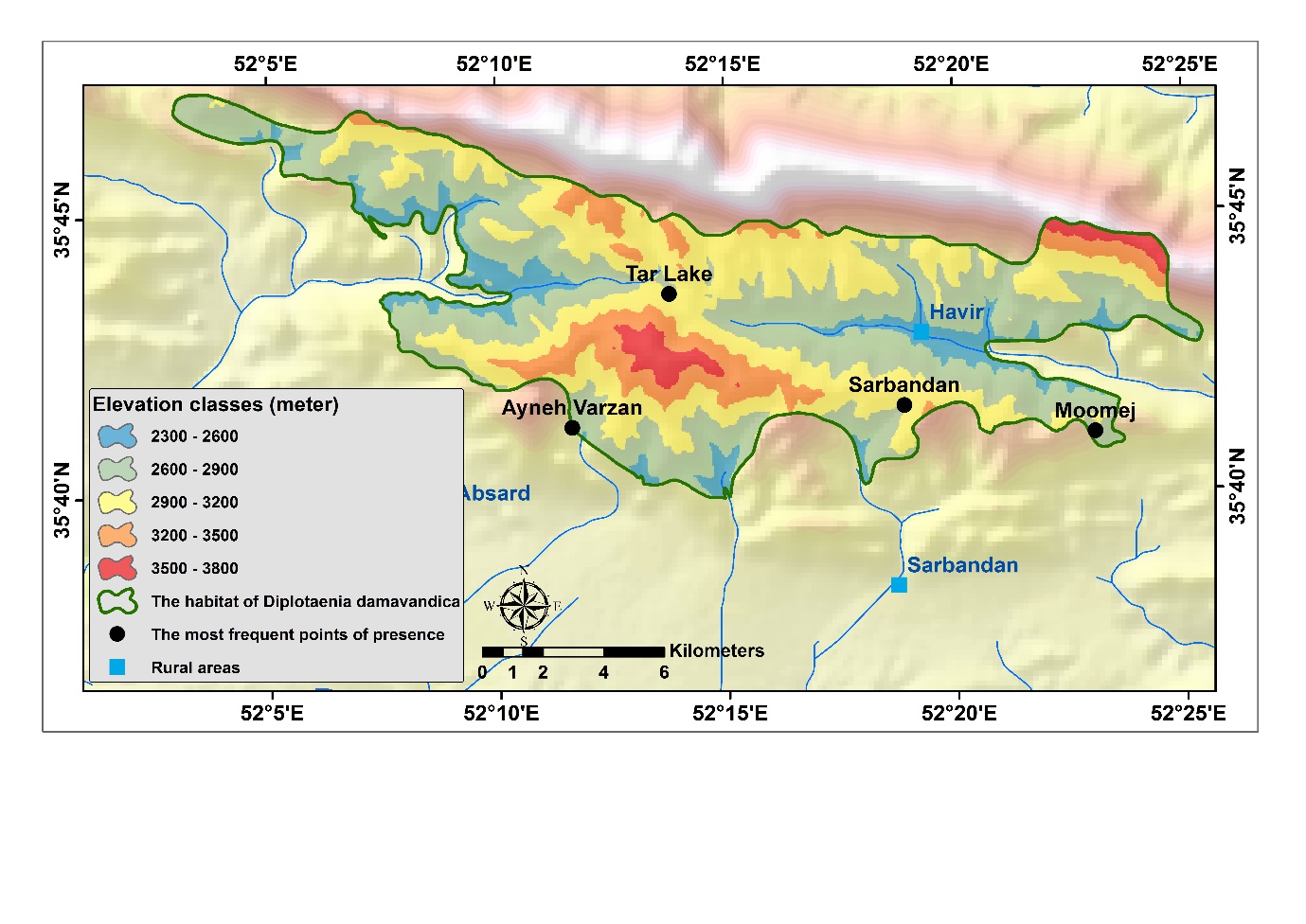


Fig. b. The elevetion classes map (m) of the study area© 2024 by Parvaneh Ashouri is licensed under CC BY 4.0 (created by ArcMap 10.5)


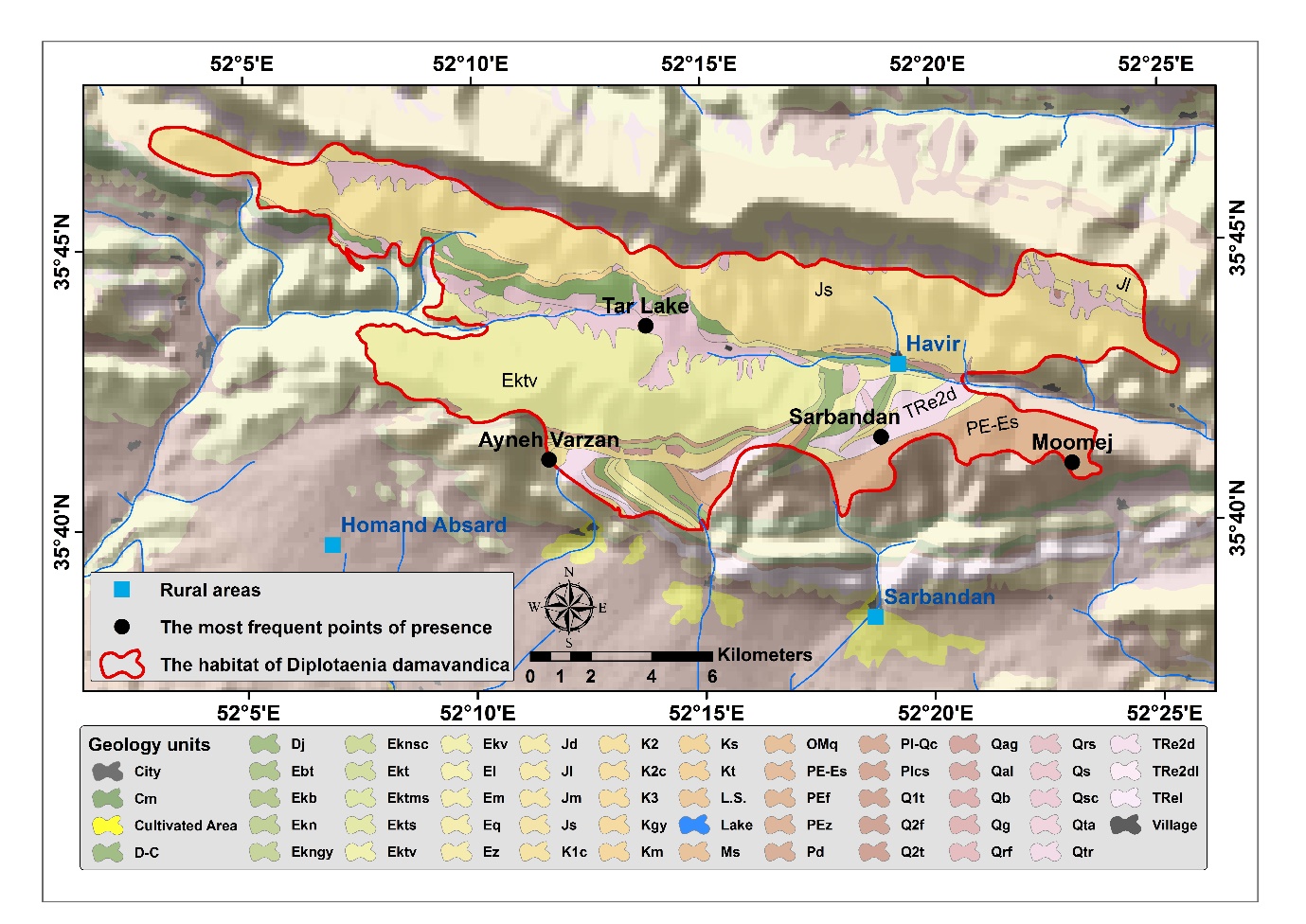


Fig. c. The geology unit map of the study area© 2024 by Parvaneh Ashouri is licensed under CC BY 4.0 (created by ArcMap 10.5)


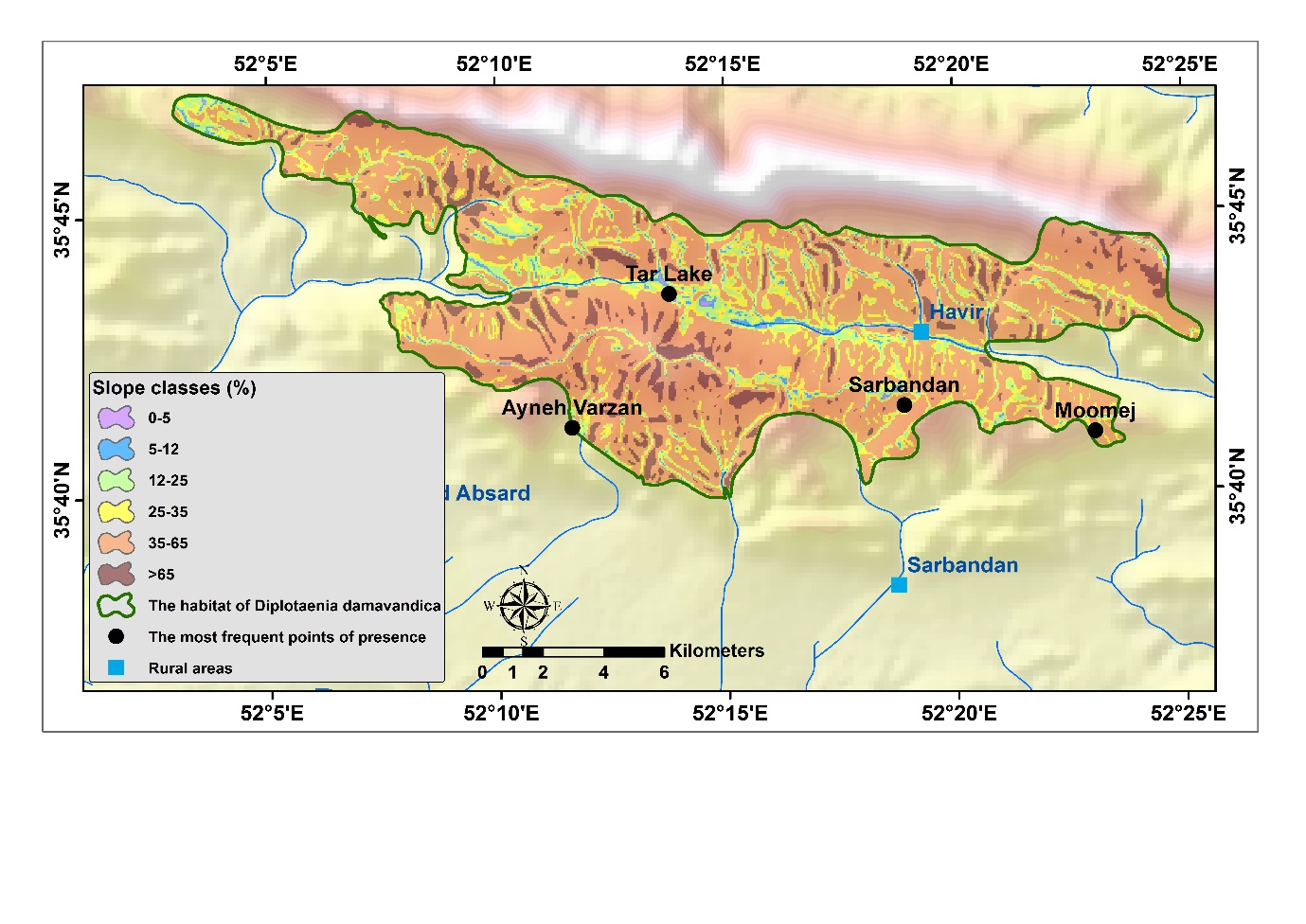


Fig. d. The slope classes (%) map of the study area© 2024 by Parvaneh Ashouri is licensed under CC BY 4.0 (created by ArcMap 10.5)
